# What Does a Positive Alzheimer Disease Blood-Based Biomarker Result Mean in People With Mild Cognitive Impairment?

**DOI:** 10.1212/WNL.0000000000214953

**Published:** 2026-05-04

**Authors:** Jemma Hazan, Kathy Y. Liu, Jeremy D. Isaacs, Nick C. Fox, Robert Howard

**Affiliations:** 1 Division of Psychiatry, University College London, London, United Kindgom;; 2 North London NHS Foundation Trust, London, United Kindgom;; 3 St George's University Hospitals NHS Foundation Trust, London, United Kindgom;; 4 Department of Psychology and Neuroscience, School of Health & Medical Sciences, City St George's, University of London, London, United Kindgom;; 5 Dementia Research Centre, Department of Neurodegenerative Disease, UCL Queen Square Institute of Neurology, University College London, London, United Kindgom; and; 6 UK Dementia Research Institute at UCL, University College London, London, United Kindgom.

## Abstract

Blood-based biomarkers (BBMs) for Alzheimer disease (AD) offer widely expanded access to biomarker-informed diagnoses in community settings that have previously lacked such resources. However, integrating them into current clinical diagnostic practice presents challenges, particularly in the diagnosis of mild cognitive impairment (MCI). Limited data are available to guide clinicians on when to test, how to counsel patients with MCI and a positive AD BBM result about their risk of dementia progression, or which evidence-based dementia prevention strategies or treatments to recommend. This Perspective article outlines the key challenges faced by clinicians and patients when addressing a diagnosis of “MCI due to AD” and offers suggestions for appropriate pretest counseling and future research directions to address current knowledge gaps.

## Introduction

Alzheimer disease (AD) can be defined biologically by the abnormal accumulation of β-amyloid (Aβ) and tau neurofibrillary tangles in the brain.^1^ The 2018 National Institute on Aging-Alzheimer's Association (NIA-AA) research framework conceptualized AD as a biological construct that exists along a continuum.^2^ AD begins with a preclinical phase, in which pathologic changes occur without objective cognitive impairment symptoms, progresses to a prodromal stage characterized by mild cognitive impairment, and finally to dementia with loss of functional independence.^3^

This reconceptualization shifts diagnosis from a purely symptom-based approach to incorporating biomarkers of molecular pathology as defining features of disease, driven by advances in biomarker technology and accessibility. CSF and PET imaging can quantify β-amyloid (Aβ) and tau pathology in vivo,^4^ while newer blood-based biomarkers (BBMs) show close correlation with CSF and PET measures in specialist settings and research cohorts with a high pretest probability of AD.^5^ Among AD BBMs, plasma phosphorylated tau-217 (p-tau217) has demonstrated superior diagnostic accuracy and specificity when compared with p-tau181 or the Aβ42/40 ratio.^5^ However, positive predictive value (PPV) and specificity are likely to be reduced in primary care populations with a lower prevalence of AD pathology,^6^ and analytic variability and comorbidities (e.g., renal impairment) highlight the need to harmonize cut-points, particularly in diverse community-based populations.^7^ Their scalable and accessible nature is likely to facilitate widespread implementation in community clinical services, which have historically lacked access to biomarker investigations,^8,9^ underscoring the need for guidance.

Mild cognitive impairment (MCI) is an umbrella clinical syndrome characterized by a change in cognitive function, objective cognitive impairment on neuropsychological testing, and preserved activities of daily living (ADLs).^10^ Both the International Working Group (IWG) 2024 recommendations^11^ and the Alzheimer's Association (AA) 2024 revised criteria^12^ support biomarker use to subtype prodromal AD, which the AA defines as conceptually equivalent to MCI due to AD. The IWG criteria further require a “common AD phenotype,” such as hippocampal amnestic syndrome, logopenic variant primary progressive aphasia, or posterior cortical atrophy.

However, in the United States, diagnoses of MCI may be made by a range of clinicians, including neurologists, geriatricians, old age psychiatrists, and more rarely primary care physicians. There is no unified national clinical guideline for MCI, and existing clinical guidance, such as the AA recommendations,^13^ remains incomplete. Although the American Academy of Neurology published updated practice guidelines in 2018,^14^ these were retired in February 2024, likely reflecting rapid advances in biomarker technologies and emerging amyloid-targeting therapies, necessitating new evidence-based guidelines.

In the United Kingdom, MCI is most frequently diagnosed by old age psychiatrists in community memory services. The National Institute for Health and Care Excellence (NICE) has no MCI-specific guidelines and, in a 2023 surveillance review, cited insufficient evidence for the use of biomarkers in this context.^15^ NICE recommends biomarkers only to support dementia subtyping when the diagnosis is uncertain and AD is suspected.^16^ By contrast, a systematic review found 8 clinical guidelines pertaining to MCI worldwide.^17^

Although MCI can be coded in diagnostic classification systems, most closely as *6D86 Mild neurocognitive disorder* in ICD-11 or *331.83 Mild neurocognitive disorder* in DSM-5,^18^ it is often used as a descriptive label in clinical practice, with no guideline-defined assessment or management pathway.

More longstanding appropriate-use recommendations exist for the investigation of MCI using PET^19^ and CSF^20^ biomarkers. These outline scenarios in which biomarker testing provides clinical value, for example, where a high PPV is needed to determine eligibility for an approved amyloid-targeting therapy, or where a strong negative predictive value can help rule out AD in cases of MCI in which AD is suspected. Recommendations for the use of AD BBMs in MCI are currently broader and less specific.^21^

As BBMs become more widely available, a cohort of individuals with MCI who test positive for AD biomarkers, a so-called “MCI due to AD”^22^ diagnosis, will likely emerge in clinical practice. This presents new challenges for clinicians, who must decide whether testing is appropriate, and how to interpret results, communicate prognostic uncertainty, and provide counseling.

The aim of this Perspective article was to review current evidence on the diagnosis, prognosis, and management of individuals with MCI due to AD and to explore ethical considerations and practical frameworks for counseling patients in this evolving diagnostic landscape.

### What Does the Diagnosis Mean?

The diagnosis of MCI remains challenging, with difficulty and inconsistency in distinguishing it from very mild dementia, particularly when interference with ADLs is subtle.^23^ Thresholds for functional impairment are open to clinical interpretation, particularly when an individual increasingly relies on compensatory adaptations, or when baseline daily activities were never performed. Consistent with this uncertainty, analyses from the National Alzheimer's Coordinating Center show large between-center variation in MCI diagnoses, with some centers classifying over 50% of assessed patients as having MCI.^24,25^

The introduction of biomarkers has the potential to help provide etiologic support for a diagnosis of MCI due to AD. However, although this is more specific than a purely clinical diagnosis of amnestic MCI,^26^ it does not exclude other potentially reversible contributors such as depression, anticholinergic medication effects, or obstructive sleep apnea, which require clinical assessment.^27^ This is consistent with IWG guidance where dysexecutive MCI is not considered primarily AD-related, even with a positive biomarker result, and alternative causes should be considered.^11^

Second, a positive AD biomarker result can influence how individuals understand and identify with their condition, transforming MCI from a descriptive clinical syndrome into a biologically defined disease state, with potential psychological and insurance implications, particularly in health care systems such the United States.^28^ These effects will depend on how the diagnosis is interpreted prognostically.

The potential drawbacks of biomarker testing in MCI also require consideration. While a positive AD biomarker result indicates an increased risk of progression to dementia, particularly in individuals expected to survive for several years, progression is not inevitable. Result disclosure may lead to anxiety and medicalization, including repeated assessments or investigations. This parallels prostate-specific antigen testing, where an elevated level can lead to invasive treatment and potential harm, despite many individuals never developing clinically significant disease. Similarly in MCI, a positive or indeterminate result may prompt further more invasive and/or expensive investigations that may not be clinically necessary. Indeed, around 20% of those tested are expected to have an indeterminate BBM result,^29^ with no clear guidance on who should have CSF or amyloid-PET confirmatory testing.^30^

AD BBM testing is inappropriate in individuals with subjective cognitive decline, in which subjective memory concerns occur without objective deficits. This position is endorsed by international recommendations on AD diagnosis.^11,13^ In this group, low pretest probability of amyloid positivity results in poor diagnostic and prognostic utility, with a low PPV and a higher false-positive rate^31^; one study reported a PPV of 50%.^32^ This increases the risk of potential psychological harm or false reassurance.

### The Natural History and Risk of Progression

Many patients want to understand their risk of progression and the likely expected disease course. A positive AD BBM result indicates (1) an increased likelihood that cognitive impairment is due to AD and (2) an altered risk of progression; however, it does not provide information about the timing or even the certainty of clinical progression for an individual. Both aspects must be interpreted in the context of age. In an older person, the PPV of a biomarker result is higher,^33^ because of increased amyloid prevalence.^34^ However, the clinical implication may be less clear because a positive result may not be directly attributable to the presenting symptoms, introducing possible diagnostic error.^7^ This is important because the fastest growing cohort of the US population includes individuals older than 80 years, with implications for increased referral to assessment services in this age group.^35^

By contrast, a negative biomarker result may be reassuring, as it suggests a low likelihood of underlying AD pathology and, therefore, a lower short-term risk of progression to AD dementia. However, a negative result does not exclude other neurodegenerative, vascular, or secondary causes of progressive cognitive impairment. This reinforces the importance of a comprehensive clinical assessment and investigations, and the danger of relying solely on an AD BBM result for diagnosis.

Cohort studies, which provide group-level rather than patient-level prognostic estimates, suggest that people with MCI have an average annual risk of progression to dementia of 10%–15%,^36^ with approximately 50% progressing to dementia over a 3-year course^37^ and a mean transition of 4 years, although trajectories vary widely.^38^ Risk stratification using biomarkers further refines these estimates: meta-analytic data indicate that the risk of progression to dementia is approximately fivefold higher among those with MCI who are both amyloid-positive and tau-positive compared with those who are biomarker-negative.^39^ Another study estimated that the lifetime risk of dementia in individuals with MCI who are positive for both amyloid and neurodegeneration ranges from 46.7% to 95.6%, depending on age.^40^ Cognitive phenotyping of patients with MCI, specifically identification of an AD phenotype before testing, significantly increases the pretest probability of amyloid positivity, the positive predictive value of plasma biomarker testing.^32^ Individuals with an AD phenotype also have a higher 3–5-year incident rate of progression to dementia compared with those with dysexecutive MCI. In one study of memory clinic patients with MCI (mean age 67 years), 40% met IWG-2 criteria for prodromal AD; their 3-year progression rate to Alzheimer-type dementia was 61%, compared with 22% among those without prodromal AD.^e1^ This illustrates how adding positive biomarkers can shift an individual from a “moderate” to a “high”-risk group, but it does not provide a definitive yes/no answer about who will develop dementia. These estimates are influenced by pretest probability (referral setting, quality of clinical and informant history, and examination) and by age and comorbidities. Biomarkers, therefore, inform the presence of underlying AD pathology and risk rather than functioning as standalone diagnostic tests. The challenge is to determine when such risk reclassification is clinically and economically worthwhile. From a patient's perspective, it may not be apparent how an increase in risk, for example, from 20%–30% to 50%–60% over a few years, would translate into changes in quality of life.

One single-center study using multivariable prediction modeling incorporated factors such as age, sex, amyloid status, APOE genotype, and Mini-Mental State Examination score to predict cognitive decline over time.^e2^ A follow-on multicenter cohort study using a larger sample and baseline MRI and CSF biomarker data validated this prediction model,^e3^ which has been incorporated into a research online app to communicate risk to patients.^e4^ Despite these advances, predictive accuracy at the individual level remains limited for MCI. Most studies derive their estimates from retrospectively defined “converter” and stable groups,^22^ but participants who drop out of longitudinal studies may have a higher risk of developing dementia than those who remain, potentially biasing results.^e5^

### What to Counsel: Will a BBM Result Change Management?

The only licensed and marketed pharmacologic treatments for MCI due to AD are the amyloid-targeting monoclonal antibodies donanemab^e6^ and lecanemab,^e7^ both of which require demonstration of amyloid pathology to confirm eligibility for treatment, thereby positioning biomarker testing not simply as supporting the diagnosis of AD but as a potential gatekeeper to treatment access. However, it remains unclear whether a standalone positive BBM would be considered sufficient to qualify for reimbursed treatment in countries such as the United States, where currently prescribing guidance recommends the use of confirmatory testing.^e8^

Symptomatic pharmacologic treatments, such as cholinesterase inhibitors, have not shown clinical efficacy in trials with participants with MCI and are not recommended for use in MCI due to AD.^e9,e10^ There are no formalized and evidenced psychosocial interventions available for this group, and in the United States, there are no structured pathways for routine follow-up to monitor disease progression.^e11^ As a result, patients are often discharged to primary care with advice to re-present if symptoms progress. For individuals with biomarker-confirmed MCI due to AD, this approach may be inadequate given their higher risk of progression, supporting a potential case for structured follow-up within specialist services for this group to ensure appropriate surveillance and counseling.

Regarding risk factor modification in MCI, clinicians typically advise general “brain-health”^e12^ behaviors such as regular physical activity, a Mediterranean-style diet, cognitive and social engagement, and vascular risk management. While these recommendations are unlikely to be harmful and may confer general health benefits, their impact on individual-level risk of progression from MCI to dementia remains unclear.^e13^ No randomized controlled trial has yet shown that lifestyle or multidomain interventions slow progression in biomarker-confirmed MCI due to AD,^e14^ and cognitive enhancement trials in cognitively unimpaired populations such as FINGER^e15^ and POINTER^e16^ cannot be directly extrapolated to individuals with established cognitive decline and positive Alzheimer biomarkers. Furthermore, the value of making a biomarker-confirmed diagnosis of MCI due to AD simply to offer lifestyle advice that applies to everyone is unclear. MCI due to AD thus occupies a hybrid space between prevention and treatment, where patient guidance should provide transparency about the current limitations in the evidence base for risk factor modification.

### How to Counsel: Disclosure and Communication

The decision to undergo AD BBM testing in MCI is complex and requires informed discussion. In the context of evidence-based treatments with modest clinical benefits, and in the absence of individually precise prognostic tools for MCI, clinicians and patients must weigh the potential benefits and harms of ascertaining biomarker status. For some, a negative test may offer reassurance, while a positive result may justify closer clinical progression monitoring or confer eligibility for clinical trial participation. Yet, uncertainty remains as to whether such investigations should be routinely performed in individuals with MCI. For example, AD biomarker testing is likely to have limited value when there is sufficient clinical evidence to support a non-AD etiology for the cognitive impairment identified on clinical assessment.

These findings highlight the importance of pretest counseling and shared decision making when deciding whether to proceed with testing. A biomarker test result may add further diagnostic and prognostic information for a patient, but it is equally important to acknowledge the potential for harm, both to patients and to the health care system, through additional costs and investigations that may not meaningfully change management.

The use of AD BBMs in MCI raises further ethical considerations. These include the potential for psychological distress, stigma, or altered self-perception after a positive result.^e17^ For some, there is a risk of increased suicidal ideation.^e18^ Nonetheless, qualitative studies suggest that patient and caregiver responses to biomarker disclosure are mixed: while some experience anxiety or pessimism,^e19^ others report relief in having an explanation for symptoms or welcome an opportunity to plan for the future.^e20^ Therefore, if biomarker testing is performed in MCI, post-test counseling is essential. There is also the potential to provide a false expectation of certainty.^e21^ It is relevant to consider whether the benefits of BBMs truly exceed the harms in all populations. As cohort age increases, the number of patients who receive a positive biomarker result but do not progress to dementia in the short term is increasingly likely to exceed the number for whom a negative result offers reassurance.

### Future Research Directions and Recommendations

A central issue is what clinicians should realistically tell patients who test positive for AD biomarkers about their prognosis, particularly considering the high prevalence of amyloid positivity in older adults. With the evidence base moving quickly, information must remain accurate and up to date. However, while personalized prognostic estimates seem valuable, synthesizing individual studies into meaningful guidance is inherently complex.

Future research should prioritize developing and evaluating structured communication and education frameworks for patients with MCI undergoing biomarker testing.^e22^ Studies assessing the psychological, behavioral, and decisional outcomes of disclosure are needed, particularly in populations with biomarker-positive MCI due to AD where uncertainty remains high. MCI due to AD counseling guidance represents a clinical priority, particularly now that the availability of AD BBMs means that confirmation of AD pathology can be made early and easily and in many more patients. Clinicians require clear, evidence-based frameworks for discussing biomarker results, and particularly the uncertainty regarding prognosis that exists in MCI. Comparative studies using vignettes or decision audits could shape understanding of how clinicians currently make testing and disclosure decisions, informing best practice.^e23^

Adequately powered secondary prevention trials that are sufficiently long enough to detect any clinically relevant effects^e24^ are also an important next step, targeting individuals with biomarker-confirmed MCI due to AD to evaluate whether lifestyle, multidomain, or pharmacologic interventions can alter disease trajectory. At the same time, consensus is needed on the definition and boundaries of MCI, as discrepancies between diagnostic frameworks (NIA-AA, IWG) and ICD definitions, as well as idiosyncrasies in clinician diagnostic practice, continue to cause confusion.

While European guidelines recommend investigating MCI to provide prognostic information,^11^ practice remains inconsistent across services. In the United Kingdom, for example, some services routinely obtain structural imaging or offer follow-up at 1 year, whereas others discharge patients after initial assessment. In the United States, a lack of a unified care pathway has similarly had consequences for heterogeneity in investigation and follow-up practices.^e11^ Establishing uniform standards for investigation and follow-up, especially in those with biomarker positivity, would improve equity and standards of care.

Further work is also required to clarify *who* should undergo testing and also *how* such testing can be most appropriately implemented. This requires both the development of formal appropriate use guidelines and a clearer understanding of the benefits and limitations of biomarker testing at a system level. This involves balancing the potential advantages of earlier etiologic diagnostic certainty and trial eligibility against costs, psychological impact, and service capacity. “Brain health clinics” have been proposed as a model for integrating prevention, diagnosis, and risk reduction^e25^ but remain controversial because they lack an evidence base.

The positioning of AD BBMs within diagnostic pathways is dependent on health care system organization and referral pathways. In many countries, including the United States and United Kingdom, primary care is the first point of contact for individuals presenting with cognitive concerns. Ongoing research is evaluating whether AD BBMs could function as a triage tool in primary care. However, there is currently insufficient evidence to support their use as first-line screening tools, given the lack of large, well-powered studies in primary care populations,^e26^ although some studies are beginning to report relevant findings.^e27^ As outlined in this article, the operationalization of these tests is nuanced and requires careful specialist assessment to ensure appropriate use.

In the United States, it is imperative that we consider the complexities of transferring biomarker-based diagnoses from specialist or academic settings into general neurology, geriatric, or nonspecialist services, where expertise in pretest and post-test counseling is more limited. The cost of BBM implementation will exceed the cost of the assay itself, comprising training, longer consultations, and potential further investigations as a result of indeterminate test results.

Currently, economic evidence is also insufficient. Studies to date report incremental cost-effectiveness ratios for BBM-based risk stratification in MCI that do not meet NICE willingness-to-pay thresholds.^e28^ Evidence that BBM-informed risk disclosure improves quality of life, through improving certainty or facilitating care planning, has yet to be quantified in quality-adjusted life year terms. Ongoing studies such as ADAPT^e29^ and READ-OUT^e30^ are, therefore, important to generate the evidence required for future policy decisions.

In the United States, cost-effectiveness arguments center on the potential for BBMs to reduce reliance on PET and CSF testing.^e31^ However, this cannot be generalized to the United Kingdom or lower-income and middle-income countries, where these investigations are not routinely used in memory services, and potential handling of indeterminate results may lead to an increased demand on confirmatory CSF or PET testing.^30^

## Conclusion

As AD BBMs become widely available in clinical practice, their application in individuals with MCI raises important questions for clinicians, health care systems, and policymakers. Across health systems, uncertainty remains about when biomarker testing should be offered, how results should be interpreted, and what prognostic advice can be given on receipt of a BBM result. Most of these studies take place in specialist tertiary settings. This is a novel and emerging technology, and we are still awaiting outcomes from larger community-based clinical studies.

In the publicly funded systems such as in the United Kingdom, the absence of specific NICE, Royal College, or professional society guidance on MCI, including MCI due to AD, contributes to uncertainty regarding testing in this context, and in the United States, guidance is nonmandatory, fragmented, and still evolving. In the United Kingdom, NICE, who conduct technology appraisals and issue guidance to the NHS, would likely say that the current absence of evidence precludes clear recommendations. In the United States, there is no equivalent technology appraisal body, but decisions regarding AD BBM use in this context are also constrained by uncertain evidence, fragmentation of guidance, and differing payer insurance policies. Further evidence on the utility of AD BBMs in memory service and specialist care settings is needed, and studies such as the ADAPT trial^e29^ are currently underway to address this.

A structured clinical pathway for MCI due to AD, with defined indications for testing and routine follow-up at 6 or 12 months, could support a more consistent and targeted use of resources. Ultimately, integrating AD BBMs into diagnostic pathways, particularly in MCI, will require the support of a robust evidence base, careful consideration of ethical issues, and wider system-level planning. Clearer national guidance would help clinicians navigate this fast-moving field, with the overarching goal of ensuring diagnostic technologies translate to improved patient outcomes.

## Glossary

AA: Alzheimer's Association
AD: Alzheimer disease
ADLs: activities of daily living
Aβ: β-amyloid
BBM: blood-based biomarker
IWG: International Working Group
MCI: mild cognitive impairment
NICE: National Institute for Health and Care Excellence
PPV: positive predictive value
